# Comparative analysis of the electroencephalogram in patients with Alzheimer's disease, diffuse axonal injury patients and healthy controls using LORETA analysis

**DOI:** 10.1590/1980-57642016dn11-020010

**Published:** 2017

**Authors:** Jéssica Natuline Ianof, Francisco José Fraga, Leonardo Alves Ferreira, Renato Teodoro Ramos, José Luiz Carlos Demario, Regina Baratho, Luís Fernando Hindi Basile, Ricardo Nitrini, Renato Anghinah

**Affiliations:** 1 Neurology Department, University of São Paulo Medical School Hospital (FMUSP-HC), São Paulo, SP, Brazil; 2 Engineering, Modeling and Applied Social Sciences Center (CECS) - Federal University of ABC (UFABC), São Paulo, SP, Brazil; 3 Psychiatry Institute - University of São Paulo, São Paulo, SP, Brazil; 4 Department of Actuarial and Quantitative Methods - Pontifical Catholic of São Paulo, São Paulo, SP, Brazil; 5 Laboratory of Psychophysiology - Methodist University of São Paulo, São Paulo, SP, Brazil

**Keywords:** dementia, Alzheimer disease, electroencephalography, brain waves, demência, doença de Alzheimer, traumatismos encefálicos, lesão axonal difusa, eletroencefalografia, ondas encefálicas

## Abstract

Alzheimer's disease (AD) is a dementia that affects a large contingent of the elderly population characterized by the presence of neurofibrillary tangles and senile plaques. Traumatic brain injury (TBI) is a non-degenerative injury caused by an external mechanical force. One of the main causes of TBI is diffuse axonal injury (DAI), promoted by acceleration-deceleration mechanisms.

**Objective:**

To understand the electroencephalographic differences in functional mechanisms between AD and DAI groups.

**Methods:**

The study included 20 subjects with AD, 19 with DAI and 17 healthy adults submitted to high resolution EEG with 128 channels. Cortical sources of EEG rhythms were estimated by exact low-resolution electromagnetic tomography (eLORETA) analysis.

**Results:**

The eLORETA analysis showed that, in comparison to the control (CTL) group, the AD group had increased theta activity in the parietal and frontal lobes and decreased alpha 2 activity in the parietal, frontal, limbic and occipital lobes. In comparison to the CTL group, the DAI group had increased theta activity in the limbic, occipital sublobar and temporal areas.

**Conclusion:**

The results suggest that individuals with AD and DAI have impairment of electrical activity in areas important for memory and learning.

## Abstract (pt)

A dooença de Alzheimer (DA) é uma demência que acomete uma grande parcela da população idosa e caracteriza-se pela presença de emaranhados neurofibrilares e placas senis. O traumatismo cranioencefálico (TCE) é uma lesão não degenerativa causada por uma força mecânica externa. Uma das principais causas de TCE é a lesão axonal difusa (LAD), causada por mecanismos de aceleração-desaceleração.

**Objetivo:**

Entender as diferenças dos mecanismos funcionais entre os grupos - DA e LAD do ponto de vista eletroencefalográfico.

**Métodos:**

Participaram deste estudo 56 indivíduos adultos. Destes, 20 com DA, 19 com LAD e 17 adultos saudáveis submetidos ao EEG de alta resolução com 128 canais. As fontes corticais dos ritmos do EEG foram estimadas pela análise por tomografia eletromagnética exata de baixa resolução (eLORETA).

**Resultados:**

A análise por eLORETA mostrou que, em comparação ao grupo controle (CTL), o grupo DA apresentou aumento da atividade teta nos lobos parietal e frontal e diminuição da atividade alfa 2 nos lobos parietal, frontal, límbico e occipital. Em comparação ao grupo CTL, o grupo LAD apresentou aumento da atividade teta nas áreas límbica, occipital sub-lobar e temporal.

**Conclusão:**

Os resultados sugerem que os indivíduos com DA e com LAD apresentam comprometimento da atividade elétrica em áreas importantes para a memória e aprendizagem.

## INTRODUCTION

Alzheimer's disease (AD) is the most common form of dementia among the elderly,^1^ accounting for 35 to 80% of cases of dementia in these individuals.^2^ It is also the most frequent cause of dementia in the Brazilian elderly population.^3^ Characterized by progressive dementia,^4^ AD is a neurodegenerative disease whose definitive diagnosis cannot yet be established without histological analysis of the brain (biopsy or necropsy). The disease is associated with specific degeneration in brain tissue, especially in pyramidal neurons, with marked intracellular presence of neurofibrillary tangles (hyperphosphorylation of β-amyloid) - resulting from the abnormal metabolism of amyloid precursor protein (APP) - in the extracellular compartment, accompanied by other structural alterations such as granulovacuolar degeneration, dendritic atrophy and loss of neural synapses.^5^ Patients with AD may have limited access to memories that shape their self-awareness and self-image, resulting in a compromised sense of identity.^6^ Over the course of the disease, other symptoms may emerge, such as disorientation, mood swings, confusion, more severe memory deficits, behavioral changes, as well as difficulties speaking, swallowing, and walking.^7^

Traumatic brain injury (TBI) is a nondegenerative and noncongenital insult to the brain from an external mechanical force. It is associated with diminished or altered state of consciousness and it can lead to permanent or temporary impairment of cognitive, physical, and psychosocial functions.^8^ The most common causes of TBI are car crashes, falls, assaults and thefts, and accidents during recreational activity.^9^ It is considered a "silent epidemic",^10^ being the major cause of morbidity and mortality^11^ and leading cause of death and sequelae in children and young adults in western industrialized countries^12^. Annually, TBI affects around 10 million people, leading to death or hospitalization.^13^

The acceleration-deceleration mechanism, responsible for diffuse axonal injury (DAI), causes the fast rotational forces attributed to shear strain, damaging the axons.^14^ This mechanism often damages the lateral and ventral regions of the frontal and temporal lobes. Deficits in attention and memory, difficulty learning new information, solving problems and planning, and problems associated with impulsivity and self-control are common sequelae.^2^ Long-term memory is usually restored, but some individuals find it difficult to learn and retain new information. Working memory is often affected at different stages - coding, storage and retrieval. These changes have a significant impact on the social and professional reintegration of the individual.^15^

Although AD and DAI have different mechanisms of injury, complaints such as memory deficits and difficulty learning new information are common to both groups of patients. For the investigation of both, electroencephalographic (EEG) recording in awake-resting state condition is an ideal low-cost and non-invasive methodology. Indeed, EEG recording has a high temporal resolution (milliseconds) that provides an optimal investigational tool for the emerging features of brain physiology.^9^ EEG procedures are well-tolerated by patients, unaffected by task difficulty and are widely available in any country. They can be repeated over time without habituation effects.^17^

When compared to groups of normal elderly subjects, AD groups are characterized by high power widespread delta and theta rhythms, as well as by low power posterior alpha and/or beta rhythms.^16-19^ Moreover, EEG amplitude modulation analysis has been shown to be useful for characterizing AD progression from mild to moderate stages.^20^

The EEG immediately after TBI initially shows epileptiform activity,^21^ followed by suppressed cortical activity - which may last from seconds to about a minute.^22^ Many patients return to normal within an hour, while others continue to present focal or generalized slowing - which can last from weeks to a few months.^23^ The theta/alpha ratio increases after mild TBI and tends to return to normal within weeks to months.^24^ Quantitative EEG also shows an immediate reduction in the mean frequency of alpha and an increase in theta slow activity. These changes usually take weeks to months to resolve. Improvement is associated with a reduction in symptoms.^23^

Low-resolution brain electromagnetic tomography (LORETA) is a mathematical algorithm that estimates the sources of EEG recorded on the scalp^25^ and is widely used in EEG studies. New improved versions of LORETA have been developed such as Standardized low-resolution brain electromagnetic tomography (sLORETA) and Exact low resolution brain electromagnetic tomography (eLORETA). sLORETA^26^ and eLORETA^27^ have the same low spatial resolution, with zero localization error, but the eLORETA provides better localization of the signal source in the presence of noise.^28^

To establish the electroencephalographic differences in functional mechanisms between Alzheimer's disease and diffuse axonal injury patients, both with memory complaints, among other shared symptoms.

## METHODS

**Ethics statement.** This study was approved by the Ethics in Research Committee (CAPPesq) of the Clinicas Hospital School of Medicine, University of São Paulo. All recruited participants provided written consent.

**Inclusion criteria.** Elderly patients diagnosed with probable AD, as determined by the National Institute of Neurological and Communicative Disorders and Stroke and the Alzheimer's disease and Related Disorders Association^29^ with CDR=1 or 2 and Mini-Mental State Examination (MMSE) scores from 13 to 29 - mild or moderate phase - were included. Subjects were recruited by the Cognitive Neurology and Behavior Group of the Neurology Department, University of São Paulo Medical School Hospital (FMUSP-HC). Patients who participated in this study had the diagnosis of AD for at least 6 months. The patients were not taking anticholinergic drugs.

Subjects aged 18 years or older diagnosed with mild/moderate (Rancho Los Amigos ≥5 and MMSE scores from 13 to 28) DAI in the chronic phase and that presented memory complaints were included. They were recruited by the Cognitive Rehabilitation Group after TBI in the Division of Neurology, Clinicas Hospital, School of Medicine, University of São Paulo. The subjects with DAI who participated in this study were examined in the chronic stage (≥6 months after TBI). Patients with DAI were diagnosed based on the following criteria:^30^

1. A loss of consciousness from the time of injury that persisted beyond 6 h;
2. No apparent hemorrhagic contusion on computed tomography (CT);
3. The presence of white matter injury on MRI.

In addition, healthy adults with no memory complaints were recruited to serve as controls. Individuals of both sexes were included in the study. The instruments used for cognitive assessment in patients and adult controls were the MMSE, verbal fluency test (VF, animals category) and clock-drawing test (CDT). Demographic data and performance on screening tests are reported in the next section.

**Exclusion criteria.** Patients using medications that can modify the EEG record (such as antidepressant drugs, tricyclic compounds, nefazodone, benzodiazepines, lithium, neuroleptics) were excluded. Subjects with other neurological and/or psychiatric disorders, or that had suffered more than one TBI were also excluded from this study.

**EEG recordings and data acquisition.** EEG signals were recorded with a digital high-resolution 128-channel device (Brain Vision) using the International 10-10 system.^17^ Sampling frequency was 10000 Hz and impedance of all electrodes was maintained below 10 kΩ. The recordings were performed at resting state, with participants comfortably seated in a reclined chair for approximately 25 min. During this period, the subjects kept their eyes closed most of the time (20 minutes). When drowsiness was noticed, they were asked to open their eyes and this event was duly noted in the EEG recording. Two neurophysiologists, who are board certified, selected the EEG tracings for further analysis.

**EEG pre-processing.** The EEGLAB software package,^31^ which runs in the MATLAB (MathWorks^®^) software environment, was used to perform all pre-processing steps. All filtering was done in the zero-phase mode. First of all, the EEG was downsampled from 10000 Hz to 1000 Hz and once again to 400 Hz after lowpass filtering to 115 Hz using a 5^th^-order Chebyshev II filter. Subsequently, two Butterworth 4^th^-order filters were applied, one to eliminate power grid interference (60 Hz, notch) and the other to remove very slow fluctuations (0.4 Hz, highpass). Eyes-opening and closing events were identified, since analysis focused on the second eyes-closed moment onwards while everything before that was discarded. Average referencing was performed and the 128-channel EEG signal was divided into 4-second epochs. Epochs with strong artifacts (outside the ±450 µV range or with slope above 250 µV/50 ms) were eliminated. Finally, ocular and muscular artifacts were removed with the EEGLAB Independent Component Analysis (ICA) tool.^31^ After all these pre-processing steps, seventy 4-second epochs for each participant were stored in .txt format to serve as input EEG files to the LORETA-KEY software (http://www.uzh.ch/keyinst/loreta).

**EEG source localization.** eLORETA was used to analyze the cortical distribution of current source density. The head model of eLORETA and the electrode coordinates are based on the Montreal Neurological Institute average MRI brain map (MNI152).^32^ The solution space was limited to the cortical gray matter, including 6239 voxels with spatial resolution of 5 cubic mm. The eLORETA tomography has been previously used in several studies.^18,33,34^ Selected artifact-free EEG fragments were analyzed to calculate the eLORETA cortical current source density from 0.5 Hz to 30 Hz. The current source density of the eLORETA cortical functioning image was calculated for eight frequency bands: delta (1.5-6 Hz), theta (6.5-8 Hz), alpha 1 (8.5-10 Hz), alpha 2 (10.5-12 Hz), beta 1 (12.5-18 Hz), beta 2 (18.5-21 Hz), beta 3 (18.5-30 Hz) and omega - full-band (1.5-30 Hz).

**Statistical analysis.** Frequency tables and descriptive statistics were used to describe the profile of the sample. The Mann-Whitney U and Kruskal-Wallis tests were used to compare the continuous variables between two or three groups, respectively. Pearson's Chi-square test was used to compare the categorical variables between the diagnostic groups. For statistical analysis, the Social Package for Social Science (SPSS) version 20 by International Business Machines (IBM) was used. The significance level adopted for the statistical tests was 5%, i.e. a p-value <0.05.

For the statistical analysis of current source density, the statistical nonparametric mapping method (SnPM)^35^ was used, available as a software tool in the LORETA-KEY package. The difference in cortical source localization between groups was assessed for each frequency band with voxel-by-voxel independent F-ratio-tests, based upon eLORETA log-transformed current source density power. Cortical voxels with significant differences were identified by means of a nonparametric randomization procedure, in the three-dimensional statistical mapping. The mean source power in each voxel and the distribution in the permutated values was compared, with threshold set at a 5% significance level. A total of 5000 data randomizations were used to determine the critical probability threshold values for the actually observed log F-ratio values, with correction for multiple comparisons across all voxels and frequencies.

## RESULTS

The final sample consisted of 17 control individuals, 20 patients with Alzheimer's disease and 19 patients with DAI. Table 1 summarizes the relevant demographic data for the CTL, AD and DAI participants. Table 2 summarizes the relevant clinical data and screening test results for the participants.

**Table 1:** Demographic data in the subgroup of Alzheimer's disease, diffuse axonal injury and healthy control participants.

Variables	CTL	AD	DAI	Statistical analyses
N	17	20	19	
Gender	-	-	-	p=0.765^a^, 0.149^b^, 0.224^c^ (Chi-square test)
Male	8	11	14	-
Female	9	9	5	-
Age	47.94 (±20.82 SE)	77.35 (±6.19 SE)	50 (±11.28 SE)	P<0.001^a,c^, 0.832^b^ (Mann-Whitney U test)
Education (years)	13.94 (±3.96 SE)	7.35 (±4.68 SE)	7.47 (±5.21 SE)	p<0.001^a,b^, 0.806^c^ (Mann-Whitney U test)

CTL: control. AD: Alzheimer's disease. DAI: diffuse axonal injury.

a CTL × AD;

b CTL × DAI;

c AD × DAI.

**Table 2:** Performance on screening tests in the subgroup Alzheimer's disease, diffuse axonal injury and healthy control participants.

Variables	CTL	AD	DAI	Statistical analyses
CDR	-	1.25 (±0.55 SE)	-	
Rancho los Amigos Scale	-	-	7 (±1.05 SE)	-
MMSE	29 (±1.59 SE)	22.55 (±3.52 SE)	23.42 (±4.44 SE)	p<0.001^a,b^, 0.359^c^ (Mann-Whitney U test)
Clock-drawing test	9.44 (±0.51 SE)	5.70 (±3.08 SE)	7.37 (±2.36 SE)	p<0.001^a,b^, 0.088^c^ (Mann-Whitney U test)
Verbal fluency (animals)	19.75 (±4.36 SE)	11.15 (±4.68 SE)	10.58 (±4.07 SE)	p<0.001^a,b^, 0.799^c^ (Mann-Whitney U test)

CTL: control; AD: Alzheimer's disease; DAI: diffuse axonal injury;

a CTL × AD;

b CTL × DAI;

c AD × DAI.

**EEG findings.** The mean alpha frequency peak was 10.23 Hz (±0.90 SE) for the CTL participants, 9.30 Hz (±0.72 SE) for the AD group and 9.73 Hz (±1.02 SE) for the DAI group. Statistical analysis (Mann-Whitney U test) was performed to test possible differences in the alpha frequency peak between the groups. A statistically significant difference was found between the CTL and AD groups (p<0.05). No statistically significant difference was found between the CTL and DAI groups (p>0.125) or between the AD and DAI groups (p>0.136).

**Figure 1:**
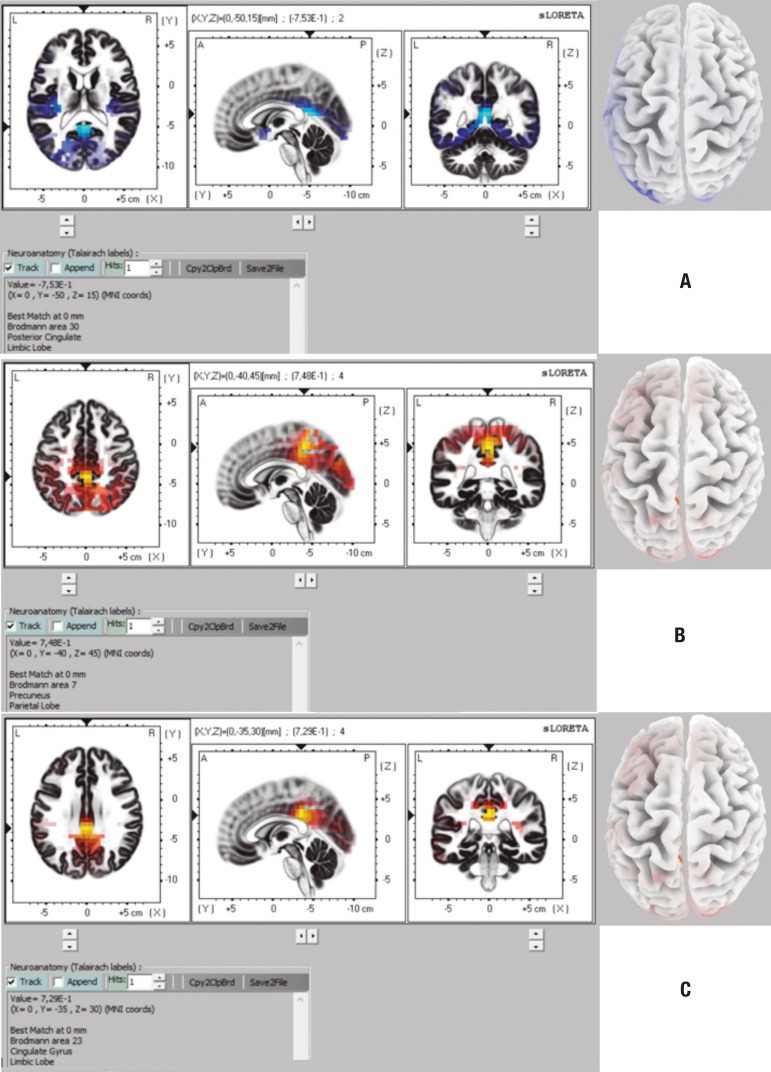
Representation of brain areas with statistically significant differences in comparison CTL × AD for theta band on 32-channel analysis (CTL<AD) [A], for alpha 2 band on 32-channel analysis (CTL>AD) [B] and for alpha 2 band on 64-channel analysis (CTL>AD) [C].

**eLORETA analysis.** The eLORETA analysis was performed with 32, 64 and 128 channels (10-20 and 10-10 system). On the comparison between CTL and AD groups, the analysis with 32 channels revealed differences in the theta and alpha2 bands. Brain structures containing voxels with statistically significant differences are shown in Table 3 and in Figure 1a and 1b. The analysis with 64 channels also revealed differences, but only in the alpha2 band. Brain structures in which voxels with statistically significant differences were found are shown in Table 4 and in Figure 1c.

**Table 3:** Brain structures with statistically significant difference on eLORETA in comparison - CTL × AD (32-channel analysis).

**Theta band**		
Lobe		Brodmann areas
Frontal		4
Parietal		1, 2, 3, 40
**Alpha 2 band**		
Lobe		Brodmann areas
Parietal		5, 7, 19, 31, 40
Frontal		5, 31
Limbic		23, 31
Occipital		7, 18, 19, 31

**Table 4:** Brain structures with statistically significant difference for alpha 2 band on eLORETA (64-channel analysis) - CTL × AD.

Lobe		Brodmann areas
Limbic		23, 31

**Figure 2:**
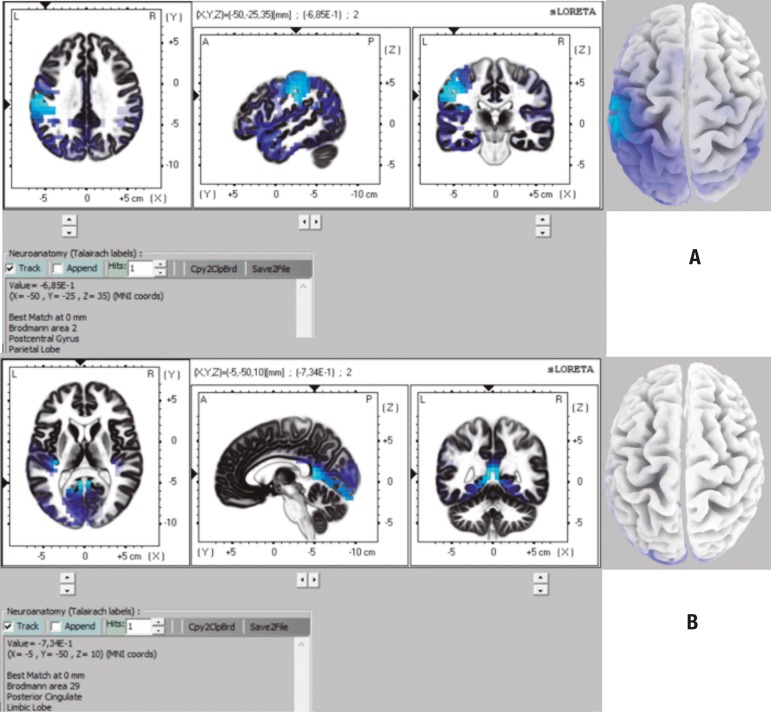
Representation of brain areas with statistically significant differences on comparison CTL × DAI for theta band on 32-channel analysis (CTL<DAI) [A], and for theta band on 64-channel analysis (CTL<DAI).

On the comparison between CTL and DAI groups, the analysis with 32 channels showed differences only in the theta band. The brain structures in which voxels with statistically significant differences were found are given in Table 5 and Figure 2a. The 64-channel analysis also showed significant differences only in the theta band (Table 5, Figure 2b).

**Table 5:** Brain structures with statistically significant difference for theta band on eLORETA in comparison - CTL × DAI.

**32 channels**	
Lobe	Brodmann areas
Limbic lobe	19, 23, 27, 28, 29, 30, 31, 34, 35, 36
Occipital	17, 18, 19, 30, 31
Sub-lobar	13
Temporal	41
**64 channels**	
Lobe	Brodmann areas
Limbic lobe	23, 27, 29, 30
Occipital	17, 18, 19, 30
Sub-lobar	13

No differences were found between the CTL and AD groups or the CTL and DAI groups for the 128-channel analysis. Also, there were no statistical differences between AD and DAI groups at any of the settings (32, 64 or 128 channels).

## DISCUSSION

Although AD and DAI patients differed in age and schooling, they had the same cognitive impairment profile, since they had many common symptoms. For this reason, we sought to compare them and determine electroencephalographic differences.

Regarding gender, no significant statistical differences were found between the CTL and AD groups or CTL and DAI groups. The DAI group had a higher number of males than the AD group, with a statistically significant difference. The literature shows that 2-3 times more men are affected by TBI than women.

The mean age of the DAI group was lower than that of the CTL group and the AD group. This was expected since TBI tends to affect younger adults whereas AD occurs mostly in elderly individuals.

The diagnosis of most cognitive disorders is clinical, but the EEG plays a role in the evaluation, classification and follow-up of these disorders. It is an important method for evaluation of cortical processing and physiological changes.^36^ A decrease in alpha and beta rhythms and increase in delta and theta frequencies are related to brain lesions and cognitive decline.^37^ Babiloni et al. (2004), using LORETA, showed that individuals with mild AD had a significant reduction in alpha 1 activity compared to healthy elderly subjects in all areas, particularly in the central, parietal, temporal and limbic areas.^38^ Gianotti et al. (2006) failed to find differences between mild/moderate AD groups and controls for the alpha 1 band. However, differences were observed for the alpha 2 band, which decreased in the occipital area more prominently in the right hemisphere.^39^ Babiloni et al. (2009) studied a group of individuals with mild cognitive impairment (MCI) and AD and found a reduction in alpha 1 activity in the occipital, temporal and parietal areas.^40^ Canuet et al. (2012) found differences between groups, using the eLORETA method, only for the alpha 1 band in the parieto-occipital region, mainly in the right pre-cuneus region.^28^ Caso et al. (2012), based on spectral analysis and sLORETA, observed that patients with AD, in comparison with controls, had lower activity for fast frequencies (alpha 1, alpha 2, beta 1 and beta 2) in the central and posterior regions.^41^ Babiloni et al. (2016) studied controls, patients with Parkinson's disease and with AD. Using LORETA, they observed that, relative to controls, patients with AD had lower alpha 1 activity in the central, parietal, occipital and temporal areas and less alpha 2 activity in the parietal and occipital areas.^42^

Our literature search found no papers using LORETA analysis in patients with DAI. Indeed, there is scant literature involving TBI and LORETA. Tomkins et al. (2011) found, using sLORETA, that TBI patients had slower delta waves than controls.^43^ The study of Corradini and Persinger (2013) used sLORETA and revealed a decrease in para-hippocampal electrical activity and in regions adjacent to the temporal lobe in individuals with mild TBI.^44^

In our study, no differences were found between the AD and DAI groups for any EEG rhythm in any configuration (32, 64 or 128 channels). One possible explanation for this is that, although the mechanisms of injury differ in the two diseases, there are many similarities in their neuroanatomy and physiology, which leads the patients to present similar symptoms. At the beginning of AD, the main characteristic of clinical features is episodic memory impairment, i.e., the patient has difficulty remembering recent events and conversations, repeats questions and stories, and has trouble finding personal objects.^45^ Decreased autobiographical memory, decreased ability to learn new information as quickly as before the TBI, less proficiency to remember faces of new acquaintances, and delayed task execution after rapid or new visual presentations, are common complaints among post-TBI patients,^44^ especially in DAI^9^.

The first pathological link between a single TBI and AD was the observation that β-amyloid plaques are present in up to 30% of patients dying in the acute phase of TBI.^46^ APP can be found accumulated in damaged axons within 2 hours after injury.^47^ Several studies have identified that the history of a single TBI is an epigenetic risk factor for the later development of clinical syndromes of cognitive impairment, such as AD.^48^ The activation of posterior and medial portions of the parietal-occipital cortex (covering the cuneus and precuneus) has been reported with considerable consistency in PET and functional MRI studies during memory tasks,^49^ specifically for success in memory recall. The onset of amnestic syndromes is related to damage in portions of the medial temporal cortex, including the hippocampus and para-hippocampal gyrus.^50^

The onset of amnestic syndromes is related to damage in portions of the medial temporal cortex, including the hippocampus and para-hippocampal gyrus (Zola-Morgan et al., 1986). At the beginning of AD, the main characteristic of the clinical feature is episodic memory impairment.

There is a pathological link between a single TBI and AD, and it is well established that TBI patients have a higher risk for developing dementia in the future. Thus, the objective of the present study was to ascertain whether these groups had similarities in EEG.

Our analyzes have shown that with fewer channels, the resolution of LORETA is improved, since a channel is responsible for a larger portion of the scalp. For this reason, the analysis using 32 channels revealed more voxels with statistical differences between the groups.

Limitations of the study include the sample size and cross-sectional methodology employed, since patients were not followed during disease progression. It should be emphasized that this study provides contributions to the national literature, since the line of research involving cognitive electroencephalography is scarce. We found no articles comparing the group of Alzheimer's disease and diffuse axonal injury in the literature with regard to electroencephalographic profile. In fact, papers investigating diffuse axonal injury are very scarce. The use of the LORETA methodology is restricted to a few research groups and existing studies generally compare dementia x controls, or two types of dementia. We found no articles involving patients with traumatic brain injury and comparing them with dementia patients.

In conclusion, our findings showed a neurofunctional similarity between AD and DAI, and that the two groups differed in relation to the controls, which was expected, since it is a comparison between pathology and normality. This neurofunctional similarity helps to understand the functionality of these diseases - our results show that the areas involved in memory and learning are compromised in both pathologies and this knowledge can be extrapolated to studies aimed at the treatment of these conditions. Drugs known to prevent memory decline in Alzheimer's disease could also be used in diffuse axonal injury patients. Also, these findings could pave the way for the use of drugs that are commonly used in the treatment of AD in patients with DAI.

Further studies on the physiology of these diseases, from an electroencephalographic perspective in a greater number of individuals are necessary, and it is also important to correlate the electroencephalographic findings and cognitive performance of patients.

## References

[1] Fratiglioni L, Launer LJ, Andersen K, Breteler MM, Copeland JR, Dartigues JF (2000). Incidence of dementia and major subtypes in Europe: a
collaborative study of population-based cohorts. Neurology.

[2] Grinberg LT, Nitrini R, Suemoto CK, Lucena Ferretti-Rebustini RE, Leite RE, Farfel JM (2013). Prevalence of dementia subtypes in a developing country: a
clinicopathological study. Clinics (Sao Paulo).

[3] Nitrini R, Caramelli P, Herrera-Jr E, Bahia VS, Caixeta LF, Radanovic M (2004). Incidence of dementia in a community-dwelling Brazilian
population. Alzheimer Dis Assoc Disord.

[4] Nizzari M, Thellug S, Corsaro A, Villa V, Pagano A, Porcile C (2012). Neurodegeneration in Alzheimer disease: role of the amyloid
precursor protein and presenilin 1 intracellular signaling. J Toxicol.

[5] James OG, Doraiswamy PM, Borges-Neto S (2015). PET Imaging of Tau Pathology in Alzheimer's Disease and
Tauopathies. Front Neurol.

[6] Haj EL, Antoine P (2017). Describe yourself to improve your autobiographical memory: A
study in Alzheimer's disease. Cortex.

[7] Winblad B, Amouyel P, Andrieu S, Ballard C, Brayne C, Brodaty H (2016). Defeating Alzheimer's disease and other dementias: a priority for
European science and society. Lancet Neurol.

[8] Jang SH (2009). Review of motor recovery in patients with traumatic brain
injury. Neurorehabilitation.

[9] Freire FR, Coelho F, Lacerda JR, Silva MF, Gonçalves VT, Machado S (2011). Cognitive rehabilitation following traumatic brain
injury. Dement. Neuropsychol.

[10] Langlois JA, Sattin RW (2005). Traumatic brain injury in the United States: research and
programs of the Centers for Disease Control and Prevention
(CDC). J Head Trauma Rehabil.

[11] Hay J, Johnson VE, Smith DH, Stewart W (2016). Chronic Traumatic Encephalopathy: The Neuropathological Legacy of
Traumatic Brain Injury. Annu Rev Pathol.

[12] McArthur DL, Chute DJ, Villablanca JP (2004). Moderate and severe traumatic brain injury: epidemiologic,
imaging and neuropathologic perspectives. Brain Pathol.

[13] Hyder AA, Wunderlich CA, Puvanachandra P, Gururaj G, Kobusingye OC (2007). The impact of traumatic brain injuries: a global
perspective. Neuro-Rehabilitation.

[14] Adams JH, Graham DI, Murray LS, Scott (1982). Diffuse axonal injury due to nonmissile head injury in humans: an
analysis of 45 cases. Ann Neurol.

[15] Vallat-Azouvi C, Weber T, Legrand L, Azouvi P (2007). Working memory after severe traumatic brain
injury. J Int Neuropsychol Soc.

[16] Babiloni C, Lizio R, Del Percio C, Marzano N, Soricelli A, Salvatore E (2013). Cortical sources of resting state EEG rhythms are sensitive to
the progression of early stage Alzheimer's disease. J Alzheimers Dis.

[17] Cassani R, Falk TH, Fraga FJ, Cecchi M, Moore DK, Anghinah R (2017). Towards automated electroencephalography-based Alzheimer's
disease diagnosis using portable low-density devices. Biomed Signal Processing and Control.

[18] Dierks T, Jelic V, Pascual-Marqui RD, Wahlund LO, Julin P, Linden DEJ (2000). Spatial pattern of cerebral glucose metabolism (PET) correlates
with localization of intracerebral EEG-generators in Alzheimer's
disease. Clin Neurophysiol.

[19] Jeong J (2004). EEG dynamics in patients with Alzheimer's disease. Clin Neurophysiol.

[20] Fraga FJ, Falk TH, Kanda PAM, Anghinah R (2013). Characterizing Alzheimer's disease severity via resting-awake EEG
amplitude modulation analysis. PLoS One.

[21] Walker AE, Kollros JJ, Case TJ (1944). The physiological basis of concussion. J Neurosurg.

[22] Shaw NA (2002). The neurophysiology of concussion. Prog Neurobiol.

[23] Nuwer MR, Hovda DA, Schrader LM, Vespa PM (2005). Routine and quantitative EEG in mild traumatic brain
injury. Clin Neurophysiol.

[24] McCrea M, Prichep L, Powell MR, Chabot R, Barr WB (2010). Acute effects and recovery after sport-related concussion:a
neurocognitive and quantitative brain electrical activity
study. J Head Trauma Rehabil.

[25] Babiloni C, Frisoni G, Pievani M, Vecchio F, Lizio R, Buttiglione M (2009a). Hippocampal volume and cortical sources of EEG alpha rhythms in
mild cognitive impairment and Alzheimer disease. Neuroimage.

[26] Pascual-Marqui RD (2002a). Standardized low-resolution brain electromagnetic tomography
(sloreta): technical details. Methods Find Exp Clin Pharmacol.

[27] Pascual-Marqui R (2007). Discrete, 3D distributed, linear imaging methods of electric neuronal
activity. Part 1: exact, zero error localization.

[28] Canuet L, Tellado I, Couceiro V, Fraile C, Fernandez-Novoa L, Ishii R (2012). Resting-state network disruption and APOE genotype in Alzheimer's
disease: a lagged functional connectivity study. PLoS One.

[29] NINCDS-ADRDA National Institute of Neurological and Communicative Disorders and
Stroke and the Alzheimer's Disease and Related Disorders
Association.

[30] Ezaki Y, Tsutsumi K, Morikawa M, Nagata I (2006). Role of diffusion-weighted magnetic resonance imaging in diffuse
axonal injury. Acta Radiol.

[31] Delorme A, Makeig S (2004). EEGLAB: an open source toolbox for analysis of single-trial
eegdynamics including independent component analysis. J Neurosci Methods.

[32] Mazziotta J, Toga A, Evans A, Fox P, Lancaster J, Zilles K (2001). A probabilistic atlas and reference system for the human brain:
International Consortium for Brain Mapping (ICBM). Philos Trans R Soc Lond B Biol Sci.

[33] Worrell GA, Lagerlund TD, Sharbrough FW, Brinkmann BH, Busacker NE, Cicora KM, O'Brien TJ (2000). Localization of the epileptic focus by lowresolution
electromagnetic tomography in patients with a lesion demonstrated by
MRI. Brain Topography.

[34] Zumsteg D, Friedman A, Wieser HG, Wennberg RA (2006). Propagation of interictal discharges in temporal lobe epilepsy:
correlation of spatiotemporal mapping with intracranial foramen ovale
electrode recordings. Clin Neurophysiol.

[35] Holmes AP, Blair RC, Watson JDG, Ford I (1996). Nonparametric Analysis of Statistic Images from Functional
Mapping Experiments. J Cerebral Blood Flow Metabol.

[36] Kanda PAM, Anghinah R, Schmidt MT, Jorge MS (2009). The clinical use of quantitative EEG in cognitive
disorders. Dement Neuropsychol.

[37] Anghinah R, Kanda PA, Lopes HF, Basile LF, Machado S, Ribeiro P (2011). Alzheimer's disease qEEG: spectral analysis versus coherence.
Which is the best measurement?. Arq Neuropsiquiatr.

[38] Babiloni C, Binetti G, Cassetta E, Cerboneschi D, Dal Forno G, Del Percio C (2004). Mapping distributed sources of cortical rhythms in mild
Alzheimer's disease. A multicentric EEG study. Neuroimage.

[39] Gianotti LR, Künig G, Lehmann D, Faber PL, Pascual-Marqui RD, Kochi K, Schreiter-Gasser U (2006). Correlation between disease severity and brain electromagnetic
LORETA tomography in Alzheimer's Disease. Clin Neurophysiol.

[40] Babiloni C, Frisoni GB, Pievani M, Vecchio F, Lizio R, Buttiglione M (2009b). Hippocampal volume and cortical sources of EEG alpha rhythms in
mild cognitive impairment and Alzheimer disease. Neuroimage.

[41] Caso F, Cursi M, Magnani G, Fanelli G, Falautano M, Comi G, Leocani L, Minicucci F (2012). Quantitative EEG and LORETA: valuable tools in discerning FTD
from AD?. Neurobiol Aging.

[42] Babiloni C, Del Percio C, Caroli A, Salvatore E, Nicolai E, Marzano N (2016). Cortical sources of resting state EEG rhythms are related to
brain hypometabolism in subjects with Alzheimer's disease: an EEG-PET
study. Neurobiol Aging.

[43] Tomkins O, Feintuch A, Benifla M, Cohen A, Friedman A, Shelef I (2011). Blood-brain barrier breakdown following traumatic brain injury: a
possible role in posttraumatic epilepsy. Cardiovasc Psychiatry Neurol.

[44] Corradini PL, Persinger MA (2013). Standardized Low Resolution Electromagnetic Tomography (sLORETA)
is a Sensitive Indicator of Protracted Neuropsychological Impairments
Following "Mild" (Concussive) Traumatic Brain Injury. J Neurol Neurophysiol.

[45] Parmera JB, Nitrini R (2015). Da investigação ao
diagnóstico. Rev Med (São Paulo).

[46] DeKosky ST, Abrahamson EE, Ciallella JR, Paljug WR, Wisniewski SR, Clark RS, Ikonomovic MD (2007). Association of increased cortical soluble abeta42 levels with
diffuse plaques after severe brain injury in humans. Arch Neurol.

[47] Johnson VE, Stewart W, Smith DH (2013). Axonal pathology in traumatic brain injury. Exp Neurol.

[48] Plassman BL, Havlik RJ, Steffens DC, Helms MJ, Newman TN, Drosdick D (2000). Documented head injury in early adulthood and risk of Alzheimer's
disease and other dementias. Neurology.

[49] Busatto G, Howard RJ, Ha Y, Brammer M, Wright I, Woodruff PW, Simmons A, Williams SC, David AS, Bullmore ET (1997). A functional magnetic resonance imaging study of episodic
memory. Neuroreport.

[50] Zola-Morgan S, Squire LR, Amaral DG (1986). Human amnesia and the medial temporal region: enduring memory
impairment following abilateral lesion limited to field CA1 of the
hippocampus. J Neurosci.

